# Swim Bladder of Farmed *Totoaba macdonaldi*: A Source of Value-Added Collagen

**DOI:** 10.3390/md21030173

**Published:** 2023-03-09

**Authors:** Honorio Cruz-López, Sergio Rodríguez-Morales, Luis M. Enríquez-Paredes, Luis Jesús Villarreal-Gómez, Conal True, Leticia Olivera-Castillo, D. Alejandro Fernández-Velasco, Lus M. López

**Affiliations:** 1 Facultad de Ciencias Marinas, Universidad Autónoma de Baja California (UABC), Carretera Transpeninsular Ensenada—Tijuana No. 3917, Col. Playitas, Ensenada 22860, Mexico; 2 Unidad de Química en Sisal, Facultad de Química, Universidad Nacional Autónoma de México, Puerto de Abrigo S/N, Sisal, Hunucma 97356, Mexico; 3 Facultad de Ciencias de la Ingeniería y Tecnología, Universidad Autónoma de Baja California (UABC), Blvd. Universitario 1000, Unidad Valle de las Palmas, Tijuana 22260, Mexico; 4 Centro de Investigación y de Estudio Avanzados del Instituto Politécnico Nacional—Unidad Mérida, Antigua Carretera a Progreso Km. 6, Merida 97310, Mexico; 5 Laboratorio de Fisicoquímica e Ingeniería de Proteínas, Departamento de Bioquímica, Facultad de Medicina, Universidad Nacional Autónoma de México, Ciudad de Mexico 04510, Mexico

**Keywords:** *Totoaba macdonaldi*, swim bladder, collagen recovery

## Abstract

Finding strategies to use the swim bladder of farmed totoaba (*Totoaba macdonaldi*) is of the utmost need to reduce waste. Fish swim bladders are rich in collagen; hence, extracting collagen is a promising alternative with benefits for aquaculture of totoaba and the environment. The elemental biochemical composition of totoaba swim bladders, including their proximate and amino acid compositions, was determined. Pepsin-soluble collagen (PSC) was used to extract collagen from swim bladders, and its characteristics were analyzed. Alcalase and papain were used for the preparation of collagen hydrolysates. Swim bladders contained 95% protein, 2.4% fat, and 0.8% ash (on a dry basis). The essential amino acid content was low, but the functional amino acid content was high. The PSC yield was high, at 68% (dry weight). The amino acid composition profile, electrophoretic pattern, and structural integrity analyses of the isolated collagen suggested it is a typical type-I collagen with high purity. The denaturalization temperature was 32.5 °C, probably attributable to the imino acid content (205 residues/1000 residues). Papain-hydrolysates (≤3 kDa) of this collagen exhibited higher radical scavenging activity than Alcalase-hydrolysates. The swim bladder from the farmed totoaba could be an ideal source to produce high-quality type I collagen and may be considered an alternative to conventional collagen sources or bioactive peptides.

## 1. Introduction

Totoaba (*Totoaba macdonaldi*, Sciaenidae) is a large croaker (up to 2 m long, and 100 kg in weight) native to the upper Gulf of California (UGC), Mexico. Once among the most valuable fisheries in the region, *T. macdonaldi* is currently listed as a critically endangered species according to Mexican law (NOM-059-SEMARNAT-2010), is included in CITES Appendix I [1], and was recently recategorized as a vulnerable species by the IUCN [2]. Originally developed for ecological purposes (conservation and restocking), totoaba aquaculture has attracted the attention of investors due to the high value of its meat [3].

The totoaba aquaculture industry in Mexico was established eleven years ago and now consists of seven licensed producers registered as Wildlife Conservation Management Units (Unidad de Manejo Ambiental—UMA) for the commercial production of totoaba (≈5 kg and 3 years old) for human consumption [3]. Current market demand is approximately 20 tons per week and is expected to increase annually (UMA-Acuario Oceanico, personal communication). Processing totoaba for meat results in by-products such as swim bladders, the market potential of which is unknown. The dried swim bladder of wild adult totoaba (known as *maw*) has characteristics similar to those of the swim bladder of wild Chinese bahaba (*Bahaba taipingensis*). Both are considered highly nutritious and valuable foods in Southeast Asia and China, and they are widely used as tonic foods in traditional Chinese medicine to improve brain function, treat insomnia and dizziness, and support postnatal recovery [4,5]. The value of totoaba *maw* in Asian markets is based on bladder size and thickness, and fish age, meaning *maws* from small totoaba would not be highly valued [5,6]. However, by-products from the processing of totoaba for meat may contain beneficial compounds (i.e., collagen from the swim bladder) with potential market value. Isolating these would help in processing waste and add value to totoaba production.

Collagen is a dominant fibrous protein in connective tissue such as skin, cartilage, bone, and other animal organs [7]. Type I collagen is widely used in the food, cosmetics, pharmaceutical, and tissue engineering industries [8]. Livestock (cattle and pigs) are the primary sources of commercial collagen. However, factors such as the fear of disease transmission (zoonoses) and the high cost of pure collagen drive a search for new and safer collagen sources [9]. Aquatic environments are seen as a promising alternative source of collagen because collagen isolated from marine and freshwater fish exhibits weak antigenicity and reduces the risk of disease transmission, among other advantages [8]. Collagen has been isolated mostly from fish skin and occasionally from swim bladders. The swim bladder of some fish species is a valuable source of bioactive compounds, mainly collagen [9,10,11]. There are various reports on preparation methods for collagen extraction from several marine and freshwater fish species and the biochemical characteristics of the extracted collagen [12,13,14,15,16]. All these collagens are Type I, although significant interspecies variation exists in collagen biochemical properties, including amino acid composition and thermal stability (denaturation temperature). The efficiency of the collagen extraction method is critical. Extraction with acidic solutions produces low yields of acid-soluble collagen (ASC). Pepsin has been used during the extraction process to increase collagen yield and decrease toxicity from telopeptides [14]. For instance, ASC and PSC were extracted from the swim bladders of miiuy croaker (*Miichthys miiuy*) [14] with yields of 1.3% (ASC) and 8.3% (PSC), and catla (*Catla catla*) [9] with yields of 22.2% (ASC) and 62.3% (PSC). Due to its higher yield, the PSC method was used in the present study.

Additionally, the fish swim bladder has been used to obtain peptides with multiple physiological activities and functions after hydrolysis with proteases [17]. For example, collagen hydrolysates from the swim bladder of the croceine croaker (*Pseudosciaena crocea*) and miiuy croaker exhibit anti-fatigue, anti-amnesic, antioxidant, and antihypertensive activities [18,19,20,21]. However, no research has been done to date on the essential biochemical composition of the totoaba swim bladder or the properties of its collagen. Therefore, clarifying the properties of the swim bladder from totoaba is essential. The objective of the present study was to isolate collagen from the swim bladder of totoaba and evaluate its composition and properties. Swim bladders were extracted from 3-year-old farmed totoaba, their biochemical composition was analyzed, PSC was isolated from them, and their physicochemical properties were evaluated. In addition, Alcalase and papain enzymatic hydrolysates were obtained, and the antioxidant activity of the peptide hydrolysate was determined.

## 2. Results and Discussion

### 2.1. Swim Bladder Biochemical Composition

The swim bladder represented 2% of the total body weight in totoaba and contained 67.43 ± 1.24% moisture. The crude protein content (dry weight) was 95.72 ± 1.07%, lipids were 2.46 ± 0.18%, and the ash was 0.88 ± 0.06%. Moisture content was low compared to other species: 75.20% in bighead carp (*Hypophthalmichthys nobilis*) [13]; 83.33% in yellowfin tuna (*Thunnus albacares*) [12]; 78.83% in miiuy croaker [14]; and 82.8% in catla [9]. These differences may be attributed to variations in swim bladder water content during tissue processing and storage, as well as to biological factors. The low total lipids and mineral contents of the totoaba swim bladder are comparable to those of the miiuy croaker [14]. Protein content was higher than reported for catla (83.0%) [9] and miiuy croaker (90.55%) [14].

Proximate composition in fish depends on many factors, including seasonal variations in feeding behavior, age, and habitat. Nineteen amino acids were identified in the totoaba swim bladders, and the amino acid profile showed collagen to be the predominant protein, which coincides with the swim bladders of other fish species [22]. Glycine was the most abundant amino acid, followed by alanine, proline, arginine, glutamic acid, hydroxyproline, and aspartic acid, which represented 85% of the total amino acids (AA). Its amino acid composition showed the totoaba swim bladder to be nutritionally poor since it contained only 12% essential AA compared to 53% conditionally essential and 35% non-essential amino acids. However, it is rich in functional AA (71%), such as glycine, glutamic acid, aspartic acid, proline, alanine, and arginine, all of which participate in and regulate key metabolic pathways [23]. It also contains high levels of hydrophobic amino acids, which are frequently found in antioxidant peptides [17]. Overall, the totoaba swim bladder had high protein and low lipid contents, suggesting it has potential use in collagen extraction, as a source of functional AA, or as a substrate for bioactive peptide production.

### 2.2. Collagen Yield

**Figure 1:**
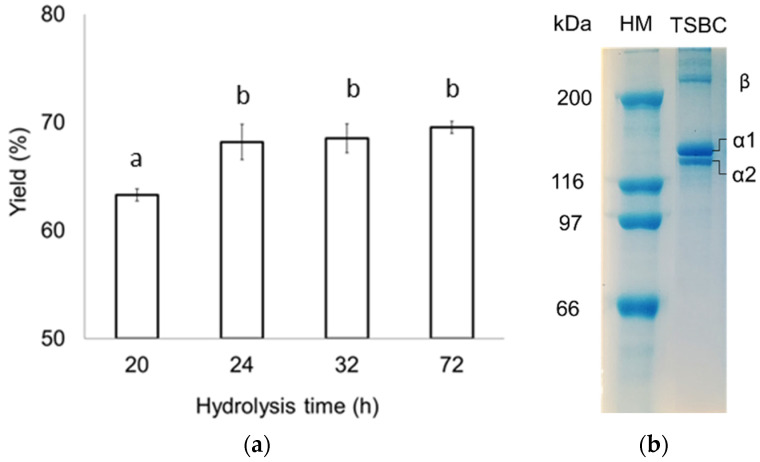
(**a**) Effect of hydrolysis time on PSC yield; (**b**) SDS-PAGE patterns of TSBC. HM: high molecular weight marker. Different letters in each row indicate significant differences (*p* < 0.05).

The hydrolysis time for PSC extraction can be as long as 72 h [13,24], so four extraction times were used in the present study: 20, 24, 32, and 72 h (Figure 1a). During the extraction process, swim bladder collagen fibers were solubilized entirely in acetic acid upon proteolysis with pepsin (24 h) with good collagen yields (68.18 ± 1.62%, dwb). According to previous studies, intermolecular cross-links in the telopeptide region and triple helices formed via condensation of aldehyde groups cause a decrease in collagen solubility [12], and the pepsin cleaves specifically on the telopeptide region, leading to isolated tropocollagen molecules. For this reason, pepsin is the principal protease used for increasing collagen extraction efficiency and reducing the collagen antigenicity caused by telopeptides [16]. Thus, the PSC yields from totoaba swim bladder are comparable to those of the Gulf corvina (*Cynoscion othonopterus*) (69%) [25] and significantly higher than the PSC yields from other species: miiuy croaker (8%), yellowfin tuna (12%), giant croaker (15%) (*Nibea japonica*), bester sturgeon (38%) (*Huso x Acipenser ruthenus*), catfish (40%) (*Tachysurus maculatus*), rohu (47%) (*Labeo rohita*), bighead carp (59%), and catla (61%) [9,12,13,14,16,26,27,28]. Interestingly, collagen yield from totoaba and other fish species is lower than the 85.3% (dwb) ASC yield reported for the seabass (*Lates calcarifer*) swim bladder [29], suggesting that the seabass swim bladder may have less cross-linked collagen fibers. These differences in collagen yield are probably due to extraction conditions, swim bladder firmness (i.e., degree of cross-linking), animal age, nutrition, and development conditions (wild or farmed). Protein content in the totoaba swim bladder collagen (TSBC) was 96.34 ± 1.19%, ash content was 0.83 ± 0.09%, and no fat was detected, which is consistent with collagen from the miiuy croaker [14].

### 2.3. Collagen Characterization

#### 2.3.1. Amino Acid Composition

All collagens have a general (Gly-X-Y)n sequence in their polypeptide chains, so glycine can be expected to be the main amino acid [7]. This agrees with the present results in that the TSBC glycine content was 309/1000 residues, followed by alanine (132/1000 residues), and proline (122/1000 residues) (Table 1). Low levels of tyrosine, histidine, isoleucine, hydroxylysine, and methionine were observed, whereas cysteine, which has been reported for collagens, was not detected [14,28]. Aromatic amino acids, mainly tyrosine, are generally found in low concentrations in PSC [28]. Compared to PSC isolated from Gulf corvina and miiuy croaker swim bladders (family, Sciaenidae) [14,25], the TSBC had higher levels of aspartic acid, glutamic acid, proline, and alanine but lower levels of valine, threonine, isoleucine, and leucine (Table 1). This variation in amino acid content could be due to various factors, such as fish species biology (health state and age), environment (water temperature and feeding), and habitat (wild or farmed). Imino acid (proline and hydroxyproline) content in the TSBC was 205/1000 residues (Table 1), which is consistent with the miiuy croaker swim bladder collagen [14]. Imino acid content has been reported to positively affect collagen structural stability because the pyrrolidine ring imposes restrictions on polypeptide chain conformation, thus strengthening the triple helix structure [30]. The degree of hydroxylation of proline (41%) and lysine (16%) also influences collagen self-assembly and stabilization [31].

#### 2.3.2. Protein Patterns

Electrophoretic analyses of the TSBC showed it to be composed mainly of two different α chains (α1 and α2), in a 2:1 proportion, and a β chain (Figure 1b). High molecular weight bands were also observed, which correspond to β-chains (dimers) and γ-chains (trimers). This suggests that TSBC is a type I collagen consisting of heterotrimeric ([α1(I)]2α2(I)) chains. The swim bladder of other fish species has been reported to contain type I collagen [10,12,30]. Using the GelAnalyzer software, the apparent molecular weights of the TSBC α1 (126 kDa) and α2 chains (116 kDa) were calculated based on migration distance. The extraction process was clearly effective because the collagen preserved its native structure. Moreover, no low molecular weight (<100 kDa) components were observed, suggesting the pepsin cleaved specifically to the telopeptide region, as previously reported [32].

#### 2.3.3. UV-Vis and Fourier Transform Infrared (FTIR) Spectroscopy

**Figure 2:**
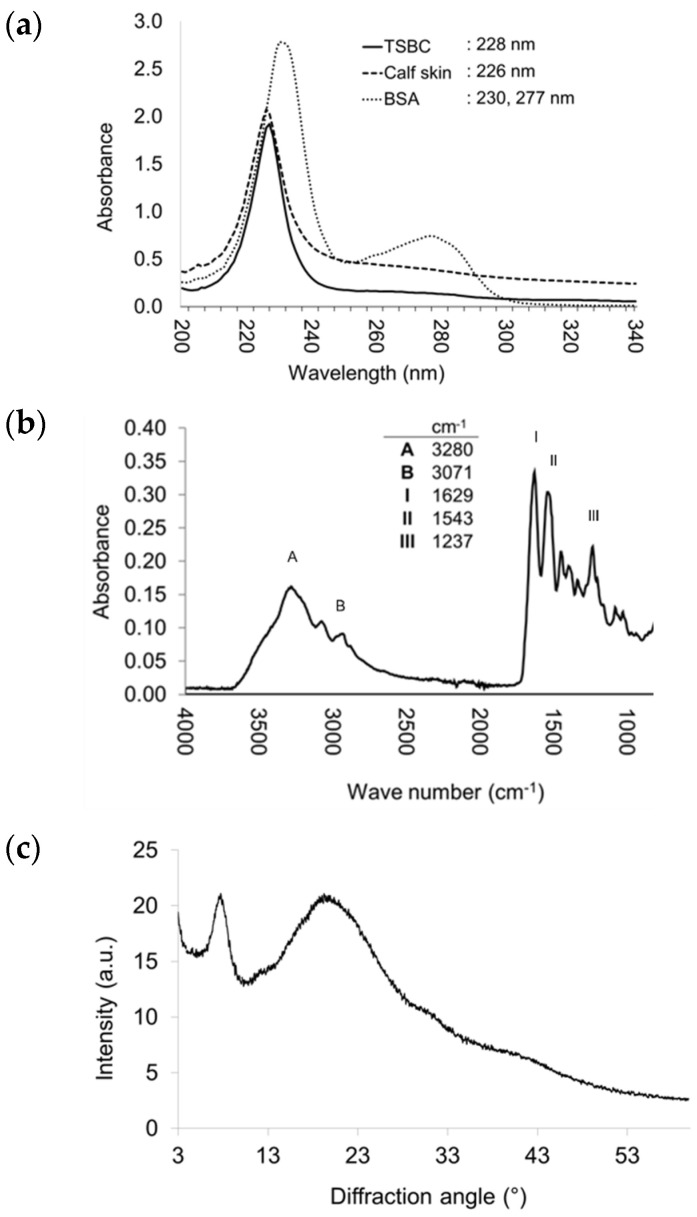
(**a**) UV spectra; (**b**) FTIR spectrum; and (**c**) X-ray diffraction spectrum (XRD) of TSBC.

Maximum absorption for collagen is near 230 nm, due to the peptide bond (R-CONH-R, amide group) of the polypeptide chains [14]. The UV-Vis spectrum is therefore an essential parameter for detecting purified collagen [14]. In this spectrum, the TSBC exhibited a maximum absorption peak at 228 nm (Figure 2a), which was similar to collagen from calf skin, grass carp (*Ctenopharyngodon idella*) [33], and miiuy croaker [14]. As expected, neither the TSBC nor the bovine serum albumin (BSA) reference exhibited a peak at 280 nm; in the TSBC, this was due to its low content of aromatic amino acids (tyrosine and phenylalanine) (Table 1). This result indicates efficient non-collagen protein elimination and consequently high TSBC purity.

In the FTIR spectrum, TSBC showed characteristic bands of amides A, B, I, II, and III (Figure 2b). Amide absorption bands A and B, which correspond to the stretching vibration of group N-H and the asymmetric stretching of CH_2_, were observed at wavenumbers of 3280 and 3071 cm^−1^, respectively. Amides I (C ═ O stretching), II (N-H bending and C-N stretching), and III (C-N stretching and N-H bending) appeared at wavenumbers 1629, 1543, and 1237 cm^−1^, respectively. The absorption ratio between amide III and the 1454 cm^−1^ wavelength was 1.05, indicating preservation of the collagen’s triple helix structure. These results coincide with those reported for collagens isolated from other fish species [9,12,13,14,16,33].

#### 2.3.4. Structural Integrity

**Figure 3:**
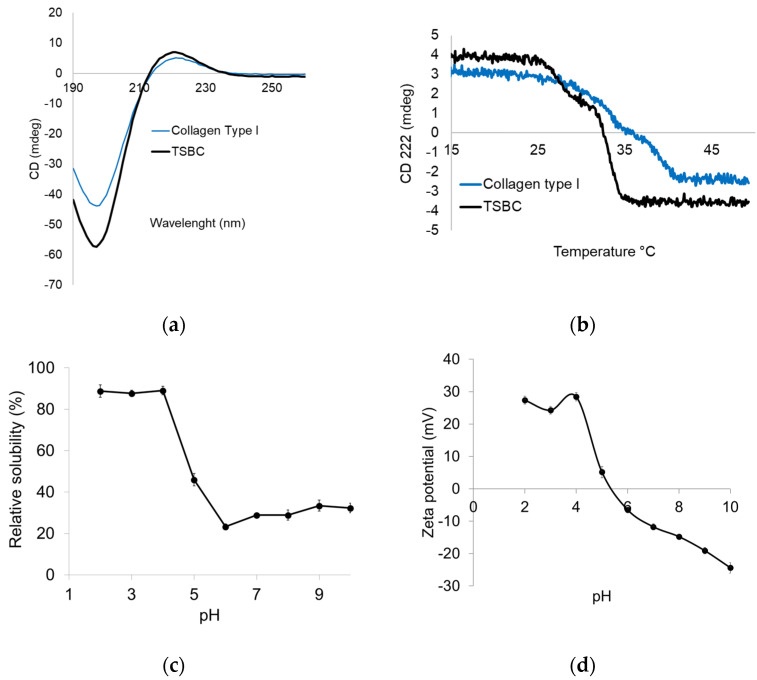
CD spectra (**a**), temperature effect on CD spectra at 221 nm (**b**), solubility (**c**), and zeta potential (**d**) of TSBC.

The TSBC XRD spectrum exhibited peaks at 7.7° and 20.02° (Figure 2c). The former was sharp and corresponded to the triple helix arrangement and distance between molecular chains; the latter was broad and corresponded to the distance between the amino acid residues along the helix [34]. Both peaks were consistent with the characteristic diffraction pattern of the collagen triple helicoidal structure [35]. The circular dichroism (CD) analysis showed the TSBC to have a weak positive absorption peak at 222 nm and a negative one at 197 nm with a crossing point (zero rotation) at 215 nm (Figure 3a). This CD spectrum pattern is characteristic of the collagen triple helix structure and consistent with previous reports [27]. The 222 nm peak disappears after thermal denaturation [27,36]. The results confirm the helix structure of TSBC remained in its native form, and therefore that the isolation process did not affect its molecular integrity.

#### 2.3.5. Thermal Unfolding

Measurements of CD molar ellipticity (θ) as a function of temperature have been used to determine the denaturation temperature (Td) [27]. The TSBS CD signal at 222 nm showed a cooperative temperature-induced unfolding transition, corresponding to the unfolding of the collagen triple helix structure, exhibiting an approximate Td of 32.5 °C (Figure 3b). Specifically, the intramolecular hydrogen bonds that stabilize the secondary structure of the collagen are disrupted, leading to a collapse of the triple helix into a random coil [37]. The present results were similar to those reported for collagen isolated from the yellowfin tuna swim bladder (33.9 °C) [12] and the Gulf corvina (32.5 °C) [25]. The Td of TSBC was higher than that of collagen from a cold-water fish such as cod (29.6 °C) [36] and a temperate-water fish such as the miiuy croaker (26.7 °C) [14]. Swim bladder collagen from marine fish remains thermostable below 35 °C, whereas in freshwater fish the unfolding midpoint is higher: 38 °C in grass carp [32] and 39.38 °C in catla [9]. Indeed, PSC isolated from the swim bladder of the freshwater fish rohu [16] retains thermal stability at up to 42.16 °C, higher than pork skin collagen (37 °C) and similar to calfskin collagen [34]. Collagen thermal behavior depends heavily on imino acid content [28], as well as the species optimum physiological temperature, which is closely related to its habitat [30]. For the farmed totoaba from UMA, average water temperature is 27 ± 1 °C, while under natural conditions, surface temperatures in the upper Gulf of California, Mexico, can range from 16 to 31 °C on the surface and from 13 to 19 °C in deep waters (from 100 to 200 m) [38]. The present TSBC thermal stability result (32.5 °C) is probably linked to the water temperature in its natural habitat. The possible use of a collagen depends heavily on its thermal stability [39], the fact that the studied TSBC has a thermal stability close to that of terrestrial mammal collagen makes it a promising alternative.

#### 2.3.6. Protein Solubility and Zeta Potential

Acid pH (2.0–4.0) caused higher solubility in the TSBC, but this parameter decreased from pH 5.0 to 6.0, resulting in protein precipitation (Figure 3c). Collagen solubility was lowest at around pH 6, but increased slightly in the pH 7.0–10.0 range. This may be due to the increased repulsion of collagen molecules as the negative charge increases [28]. Similar results have been reported for PSC in the swim bladders of grass carp [33], miiuy croaker [14], Gulf corvina [25], and giant croaker [28]. Zeta potential is a key marker of colloidal dispersion stability and varies in response to pH [14]. As pH increased in the TSBC suspension, the zeta potential progressively decreased from +27 mV (pH 2) to less than −24 mV at pH 10 (Figure 3d). At a high potential magnitude (positive or negative), a solution will resist aggregation, whereas low potential tends to lead to the formation of aggregates. For TSBC, the zero-surface net charge occurred at pH 5.4; this is considered the isoelectric point (pI) and is consistent with the protein solubility results (Figure 3c). Since the pI occurred at an acid pH, it may be associated with higher contents of glutamic acid and aspartic acid rather than of basic amino acids, such as histidine, lysine, and arginine (Table 1). The pI value was lower than that reported for swim bladder collagen from the miiuy croaker (6.85) [14] but similar to that of yellowfin tuna (5.93) [12]. In collagen, the pI is generally closely linked to the amino acid composition distribution on its surface.

### 2.4. Collagen Hydrolysate

**Figure 4:**
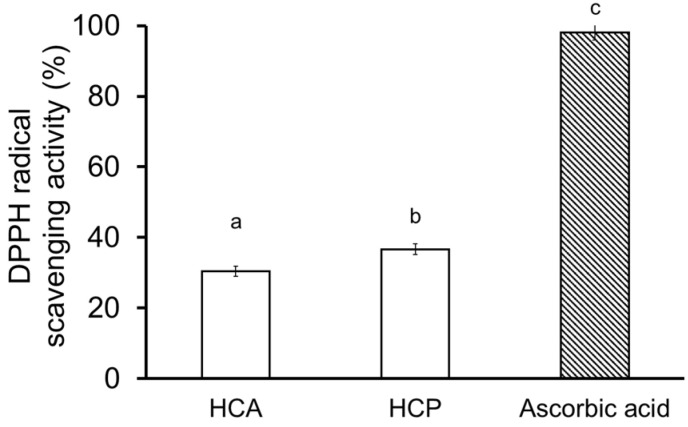
DPPH radical activities of HCA and HCP (3.2 mg mL^−1^). Different letters in each row indicate significant differences (*p* < 0.05).

The totoaba swim bladder has putatively positive therapeutic effects in traditional Chinese medicine [6]. Peptides and collagen from croaker swim bladders have been shown to remove free radicals [17,20,21]. The DPPH radical scavenging assay is a popular and efficient way of predicting antioxidant activity since the DPPH radical is more stable than hydroxyl and superoxide radicals [19]. Using the DPPH assay, the antioxidant activity of ultrafiltered fractions (≤3 kDa) of the collagen hydrolysates was tested at 3.2 mg mL^−1^. Antioxidant activity was 37% higher (*p* < 0.05) with the HCP than the HCA, although ascorbic acid far exceeded both (Figure 4). This contrasts with the antioxidant activity reported for hydrolysates from the swim bladder of the croceine croaker and the miiuy croaker, in which, at 15–25 mg protein mL^−1^, the Alcalase^®^ hydrolysate had significantly higher activity than hydrolysates prepared with papain, pepsin, neutrase, and trypsin [17]. As an additional finding, after ultrafiltration, a lower concentration of HCA and HCP (3.2 mg mL^−1^) produced a higher antioxidant activity than in the above study. Overall, the present partial antioxidant activity indicates that this parameter depends strongly on the enzyme used for hydrolysis, suggesting further research is needed to isolate active peptides and clarify their antioxidant activity.

## 3. Materials and Methods

### 3.1. Chemicals

Pepsin from porcine stomach mucosa, dialysis membrane (14 kDa MWCO), DPPH, and type I collagen standard solutions from calf skin were purchased from Sigma-Aldrich (St. Louis, MO, USA). Alcalase^®^ 2.4 L (a proteinase from *Bacillus licheniformis*) was donated by Novozymes (Mexico City, Mexico). Papain enzyme from *Carica papaya* (30,000 U/mg) and Amicon ultrafiltration tubes (3 kDa MWCO) were purchased from Merck Corporation (Burlington, MA, USA). The protein marker and bovine serum albumin standard (2 mg mL^−1^) were obtained from Bio-Rad Laboratories (Hercules, CA, USA). Solvents for amino acid analysis were HPLC-grade (T.J. Baker, Chemicals, PA, USA). All other chemicals used in this investigation were of analytical grade and used as received.

### 3.2. Swim Bladder Collection and Preparation

Totoabas were provided by the UMA (UBP) of the Facultad de Ciencias Marinas (FCM), Universidad Autónoma de Baja California (UABC), Mexico, where this study was performed. A total of 24 fish (average body weight = 2.57 ± 0.264 kg; 3-year-old) were randomly sampled from three, eight-thousand liter tanks (i.e., 8 fish/tank). The tanks were supplied with continuously recirculated seawater at a flow rate of 1.6 L min. During cultivation, the physical and chemical water parameters were monitored twice daily to maintain the recommended conditions for totoaba culture. The temperature was controlled at 27 ± 1 °C with thermo-control of chillers. The salinity was measured with a refractometer and maintained at an average of 35 ± 0.5‰. The photoperiod was set at a 12:12 light:dark ratio. The oxygen concentration was kept higher than 6 mg L^−1^. Before feeding, total ammonia-nitrogen (NH_4_-N) and total nitrite-nitrogen (NO-N) were measured daily with colorimetric test kits (Aquarium Pharmaceutical, Mars, PA, USA) and maintained below 0.2 and 0.1 mg L^−1^, respectively.

Fish were sedated using a clove oil solution (40 mg L^−1^) and euthanized by pithing to avoid distress and suffering, following applicable national animal welfare guidelines (NOM-033-ZOO-1995). The fish were dissected to manually remove the swim bladder, which was transported under refrigeration (3 ± 1 °C) to the Aquaculture Nutrition Laboratory. Processing of the byproducts represented 53% of the total fish weight, and the swim bladder accounted for approximately 11% of the total byproduct. Blood vessels and residual fat attached to the swim bladder were removed manually, the bladders cleaned, and cut into small pieces (0.4 kg/bag). These pieces were used in the proximate analysis and collagen extraction.

### 3.3. Proximate Analysis

Swim bladder moisture, protein, and ash contents were quantified using established methods [40]. Moisture content was measured by weight difference after drying (105 °C for 12 h) and ash content by combustion in a furnace at 550 °C for 12 h. Total lipids were measured using a modification of the Folch extraction method, replacing chloroform with less toxic dichloromethane [41]. Total nitrogen content was measured with the Kjeldahl method in a Vapodest 450 analyzer (Gerhardt Analytical Systems Co., Königswinter, Germany). Crude protein was calculated using a 6.25 conversion factor.

### 3.4. Amino Acid Analysis

The swim bladder amino acid composition was analyzed with the PicoTag method (Waters Corp., Milford, MA, USA). Samples were hydrolyzed with 6 N hydrochloric acid and 0.1% phenol and incubated in a nitrogen atmosphere at 110 °C for 22 h. After hydrolysis, samples and standards were derivatized with the phenyl isothiocyanate (PITC) reagent and reconstituted in a sodium phosphate buffer (5 mM, pH 7.4) containing 5% (v/v) acetonitrile. The derivatives were analyzed by reverse-phase chromatography (RP-UHPLC) in an Ultimate 3000 UHPLC system (Thermo Scientific, Waltham, MA, USA) with Chromeleon software 7.2 (Chromatography Data System). Five µL of the samples were injected into a Pico-Tag^®^ C18 column (3.9 mm × 150 mm, 4 µm and 60 Å). Separation of the amino acids was done with a binary gradient using an AccQ-Tag eluent as the mobile phase A (Waters Inc.) and aqueous acetonitrile as phase B (60% (*v*/*v*) in water) in the following gradient mode: 0.0% B at 0.0 min; 46% B for 10 min; then 100% B at 10.5 min; 100% B at 12 min; and returning to 0.0% B at 12.5 min. The total run time was 22 min with a 1.0 mL min flow rate. The column temperature was set at 38 °C, and UV detection was performed at 254 nm, which was used for calculation. Amino acid identification and quantitation were performed using a standard amino acid mixture as a reference.

### 3.5. Swim Bladder Collagen Isolation and Purification

#### 3.5.1. Pre-Treatment

Collagen extraction was performed using the protocol previously described [13,25]. All pre-treatment steps were performed at 4 °C under gentle continuous stirring for 12 h. The swim bladders were pre-treated to remove non-collagenous proteins, pigments, fats, and other impurities. The swim bladders were submerged in 0.1 M NaOH, kept at a 1:20 ratio (*w*/*v*), and then washed with cold distilled water until all the alkaline solution was eliminated. The tissue was degreased with 10% (*v*/*v*) *n*-butanol at a 1:20 (*w*/*v*) sample/solvent ratio. Both the alkaline wash and degreasing steps were performed by changing solutions at 4 h intervals.

#### 3.5.2. Collagen Extraction and Purification

The pre-treated swim bladders were digested in 0.5 M acetic acid containing 2% pepsin (*w*/*w*) at a 1:40 (*w*/*v*) tissue/solution ratio. The mixture was continuously stirred at 4 °C for 24 h. After digestion, the viscous extract was filtered with two layers of cheesecloth and precipitated by adding NaCl to a final concentration of 1.2 M. The precipitate was collected by centrifuging at 16,000× *g* at 4 °C for 20 min using a Megafuge 16R centrifuge (Thermo Scientific Co., Waltham, MA, USA), and the resulting pellets were dissolved in 0.5 M acetic acid. This solution was purified using a dialysis membrane (14 kDa molecular weight cut-off) against distilled water for 72 h with a change of solution every 4 h. The resulting collagen was lyophilized (Free Zone 2.5 L, Labconco Corp., Kansas, MO, USA) and stored at −20 °C until further analysis.

The collagen yield was calculated based on the wet and dry weights of the raw material before and after processing, using Equation (1).

(1) $$\mathrm{Yield}\;\left(\%\right)=\left[\frac{\mathrm{Wc}\;\left(g\right)}{\mathrm{Wd}\;\left(g\right)}\;\right]\;\times \;100$$

where Wc is the weight of the lyophilized collagen and Wd is the dry weight of the initial swim bladder prior to pre-treatments.

### 3.6. Collagen Characterization

#### 3.6.1. Amino Acid Composition

The amino acid composition of the lyophilized collagen was analyzed as described in Section 2.3.1. Amino acid quantification was expressed as the number of residues per 1000 total residues. The hydroxylation of proline (Pro) and lysine (Lys) was calculated from the amino acid composition using Equations (2) and (3):

(2) $$\mathrm{Degree}\;\mathrm{of}\;\mathrm{Pro}\;\mathrm{hydroxylation}\;\left(\%\right)=\left[\frac{\mathrm{Hydroxyproline}\;\mathrm{content}}{\mathrm{Hydroxyproline}\;\mathrm{content}+\mathrm{Proline}\;\mathrm{content}}\;\right]\times 100$$

(3) $$\mathrm{Degree}\;\mathrm{of}\;\mathrm{Lys}\;\mathrm{hydroxylation}\;(\%)=\left[\frac{\mathrm{Hydroxylysine}\;\mathrm{content}}{\mathrm{Hydroxylysine}\;\mathrm{content}+\mathrm{Lysine}\;\mathrm{content}}\;\right]\times 100$$

#### 3.6.2. Electrophoretic Pattern

Collagen molecular weight (MW) was determined using sodium dodecyl sulfate polyacrylamide gel electrophoresis (SDS-PAGE) according to the Laemmli method [42]. Briefly, the lyophilized collagen was dissolved in 0.1 M acetic acid and mixed at a 1:2 (*v*/*v*) ratio with sample buffer (0.5 M Tris-HCl, pH 6.8; containing 5% SDS, 20% glycerol, 5% β-ME, and 0.2% bromophenol blue). The mixed solution was incubated at 95 °C for 5 min. A 10 μL sample was processed using discontinuous polyacrylamide gel electrophoresis (7.5% separator and 4% stacking). A molecular weight protein marker was used to estimate collagen MW. The electrophoresis analysis was run at a 25 mA constant current voltage using a Mini-Protean apparatus (Bio-Rad Laboratories, Watford, UK). Protein bands were stained using Coomassie Brilliant Blue R250 solution.

#### 3.6.3. Ultraviolet Measurements

Spectra measurements were performed using a Multiskan GO spectrophotometer (Thermo Scientific). The lyophilized collagen was dissolved in 0.1 M acetic acid at a concentration of 0.1 mg mL^−1^, under continuous stirring at 4 °C for 12 h. The sample solution was placed in a quartz cell with a 10 mm path length. The UV spectrum was measured at wavelengths between 200 and 450 nm. The baseline was set at 0.1 M acetic acid, and the control standards (bovine serum albumin and collagen type I from calf skin) were run under the same conditions.

#### 3.6.4. FTIR Analysis of Functional Groups

The FTIR spectra for collagen were measured using a Thermo Nicolet Nexus 670 FTIR spectrometer (Thermo Scientific). Lyophilized collagen (5 mg) was mixed with 100 mg of dried potassium bromide (KBr) and compressed under dry conditions. A salt disc was inserted into the sample holder and scanned 40 times from 4000 to 400 cm^−1^ with a resolution of 2 cm^−1^ and compared to a background spectrum recorded from the empty cell at room temperature. The results were plotted between absorbance and wavenumber (cm^−1^).

#### 3.6.5. X-ray Diffraction and Circular Dichroism

The collagen crystal structures were determined using an X-ray diffraction instrument (Bruker D8 Advance DaVinci, Karlsruhe, Germany) equipped with CuKα radiation (λ = 1.5406 Å), a 40 kV tube voltage, and 40 mA of current. The scans were recorded in the 2θ (2 theta) range between 3 and 60° at 0.02°/s steps. The CD spectrum was recorded to quantify the preservation of the collagen secondary structure. The collagen solution was placed in a quartz cell (10 mm), and CD spectra were measured using a Chirascan spectropolarimeter (Applied Photophysics Ltd., Leatherhead, UK). The lyophilized collagen was dissolved in 0.1 M acetic acid at a concentration of 0.1 mg mL^−1^ and continuously stirred in a cold room (8 °C) for 24 h. Collagen solutions were then placed in a quartz cell with a 0.1 cm path length. The CD spectra were recorded between 190 and 260 nm at 15 °C at a 50 nm/min scan speed with a 1.0 nm interval. The temperature of the sample was controlled using a Peltier device (Quantum-Northwestern) and measured with a thermocouple inside the cuvette. Collagen Td was measured by measuring ellipticity at a fixed wavelength of 222 nm within the 15–50 °C temperature range at a 1 °C/min heating rate.

#### 3.6.6. Protein Solubility

Solubility was measured following an established method [28]. Briefly, lyophilized collagen was dissolved in 0.1 M acetic acid to a final concentration of 3 mg mL^−1^. The mixture was stirred for 3 h at 4 °C and centrifuged at 15,000× *g* for 15 min (Megafuge 16R, Thermo Scientific). The supernatant pH was adjusted (1M NaOH or HCl) to obtain a final pH ranging from 2 to 10 (a final volume of 5 mL) and centrifuged at 15,000× *g* for 15 min at 4 °C. The supernatant protein concentration was determined based on the Bradford method. BSA was used as the standard and control. Relative solubility was calculated by comparison with the solubility obtained at the pH exhibiting the highest solubility.

#### 3.6.7. Zeta Potential

Lyophilized collagen was dissolved in 0.1 M acetic acid to a final concentration of 0.1 mg mL^−1^ and the mixtures were continuously stirred at 4 °C for 24 h. The pH of the collagen solution was adjusted to a 2–10 range using NaOH and HCl (1M). A one milliliter collagen solution was transferred to a capillary cell, and the collagen zeta (ζ) potential was measured using a zeta potential analyzer (Zetasizer Nano ZS90, Malvern Instr., Malvern, UK). The isoelectric point was identified.

### 3.7. Collagen Hydrolysate

#### 3.7.1. Enzymatic Hydrolysis

The collagen hydrolysate was prepared by first dissolving the collagen in ultrapure water (1:30, *w*/*v*) and denaturing it at 50 °C for 10 min in a water bath. Hydrolysis conditions were 50 °C and pH 8 for Alcalase^®^ and 50 °C and pH 7 for papain. Hydrolysis was initiated by adding protease to the mixture at a 2% (*w*/*w*) E/S ratio. Enzymatic hydrolysis was done in a water bath at 50 °C for 5 h. After incubation, the enzymes were inactivated by heating the sample to 95 °C for 10 min, and the undigested collagen was precipitated by centrifuging at 10,000× *g* for 10 min at 4 °C. The supernatant of both hydrolysates was collected and ultrafiltered in an Amicon ultrafiltration unit (Merck Inc., Burlington, MA, USA) with a 3 kDa molecular weight cutoff (MWCO). The ultrafiltered fraction (<3 kDa) was collected, lyophilized, and labeled as Alcalase^®^ (HCA) or papain (HCP) collagen hydrolysate.

#### 3.7.2. DPPH Radical Scavenging Activity

The DPPH (2,2-diphenyl-1-picrylhydrazyl) scavenging method was applied according to Lee [43]. Briefly, samples were dissolved in deionized water at 3.2 mg mL^−1^, and a 50 μL sample was mixed with 50 μL of 0.120 mM DPPH in a 96-well microplate. This solution was mixed vigorously and left to stand at room temperature in darkness for 30 min. Sample absorbance was measured at 517 nm using a Multiskan GO microplate spectrophotometer (Thermo Scientific). Ascorbic acid was used as the reference. The percentage of DPPH radical scavenging activity was calculated using Equation (4):

(4) $$\mathrm{DPPH}\;\mathrm{scavenging}\;\mathrm{activity}\;(\%)=[(\;A_{0}\;-\;A_{1})/A_{0}]\;\times \;100$$

where A_0_ is DPPH solution absorbance and A_1_ is sample absorbance.

### 3.8. Statistical Analysis

The data were expressed as the mean ± standard deviation of the three replicates. All statistical analyses were run with the STATISTICA software (Version 12, TIBCO Software Inc., Palo Alto, CA, USA).

## 4. Conclusions

To our knowledge, this study is the first report on the elemental biochemical composition, isolation, and characteristics of collagen from the swim bladder of farmed totoaba (*T. macdonaldi*). The totoaba swim bladder has high protein and low lipid contents, suggesting that it is a possible food supplement with health benefits. The collagen yield was high (68%). The amino acid composition and protein pattern were typical of type-I collagen ([α1(I)]2α2(I)). Structural integrity analyses confirmed that the extraction process used here preserved the native collagen triple helix structure with high purity. The extracted collagen exhibited good thermal stability (32.5 °C), which correlated with its imino acid content. The antioxidant activities of the collagen hydrolysates were influenced by the enzyme used, and further research should be done to purify and identify antioxidant peptides. The present results constitute a baseline for future studies on the production of bioactive peptides and biomedical applications for this collagen. The results can also help promote the use of the swim bladder from farmed totoaba as an alternative to conventional collagen sources or as a functional food, which will reduce by-product generation and provide added value to the culture of totoaba.

## Figures and Tables

**Table 1:** Amino acid composition of the swim bladder (composition percentage) and TSBC (residues/1000 residues).

Amino Acids	Swim Bladder	TSBC	PSC Miiuy Croaker ^1^
Asx	5.13 ± 0.05	52 ± 1.48	39
Glx	9.61 ± 0.14	101 ± 1.95	85
Hyp	5.43 ± 0.05	83 ± 1.43	88
Ser	2.02 ± 0.08	23 ± 0.69	28
Gly	29.19 ± 0.31	309 ± 3.15	334
His	0.47 ± 0.02	5 ± 0.34	8
Arg	11.58 ± 0.37	59 ± 4.47	55
Thr	1.76 ± 0.06	13 ± 2.62	22
Ala	12.26 ± 0.13	132 ± 1.27	95
Pro	12.10 ± 0.13	122 ± 1.33	112
Tyr	0.47 ± 0.03	2 ± 0.19	2
Val	1.58 ± 0.04	16 ± 0.30	33
Met	1.36 ± 0.03	7 ± 0.47	5
Cys	0.03 ± 0.01	Not detected	0.4
Ile	0.63 ± 0.01	5 ± 0.22	13
Leu	1.94 ± 0.05	20 ± 0.08	27
Hyl	0.28 ± 0.02	5 ± 0.22	6
Phe	1.61 ± 0.03	19 ± 0.54	23
Lys	2.52 ± 0.04	31 ± 1.63	24
Imino acid ^2^		205	199.5
Degree of Hydroxylation (%)			
Pro		40.65	
Lys		14.43	

^1^ PSC from miiuy croaker (*M. miiuy*) [14].^2^ Imino acids: proline + hydroxyproline.

## Data Availability

Relevant information has been added to the article.
